# Identification of key module and hub genes in pulpitis using weighted gene co-expression network analysis

**DOI:** 10.1186/s12903-022-02638-9

**Published:** 2023-01-02

**Authors:** Denghui Zhang, Chen Zheng, Tianer Zhu, Fan Yang, Yiqun Zhou

**Affiliations:** Stomatology Hospital, School of Stomatology, Zhejiang University School of Medicine, Zhejiang Provincial Clinical Research Center for Oral Diseases, Key Laboratory of Oral Biomedical Research of Zhejiang Province, Cancer Center of Zhejiang University, Hangzhou, 310006 China

**Keywords:** Pulpitis, Bioinfomatics, Pulp capping, Hub genes, Weighted gene co-expression network analysis

## Abstract

**Background:**

Pulpitis is a common disease mainly caused by bacteria. Conventional approaches of diagnosing the state of dental pulp are mainly based on clinical symptoms, thereby harbor deficiencies. The accurate and rapid diagnosis of pulpitis is important for choosing the suitable therapy. The study aimed to identify pulpits related key genes by integrating micro-array data analysis and systems biology network-based methods such as weighted gene co-expression network analysis (WGCNA).

**Methods:**

The micro-array data of 13 inflamed pulp and 11 normal pulp were acquired from Gene Expression Omnibus (GEO). WGCNA was utilized to establish a genetic network and categorize genes into diverse modules. Hub genes in the most associated module to pulpitis were screened out using high module group members (MM) methods. Pulpitis model in rat was constructed and iRoot BP plus was applied to cap pulp. Reverse transcription-quantitative polymerase chain reaction (RT-qPCR) was used for validation of hub genes.

**Results:**

WGCNA was established and genes were categorized into 22 modules. The darkgrey module had the highest correlation with pulpitis among them. A total of 5 hub genes (*HMOX1, LOX, ACTG1, STAT3, GNB5*) were identified. RT-qPCR proved the differences in expression levels of *HMOX1, LOX, ACTG1, STAT3, GNB5* in inflamed dental pulp. Pulp capping reversed the expression level of *HMOX1, LOX, ACTG1*.

**Conclusion:**

The study was the first to produce a holistic view of pulpitis, screen out and validate hub genes involved in pulpitis using WGCNA method. Pulp capping using iRoot BP plus could reverse partial hub genes.

**Supplementary Information:**

The online version contains supplementary material available at 10.1186/s12903-022-02638-9.

## Introduction

Inflammation of tooth pulp is known as pulpitis. As one of the most common dental diseases, pulpitis is triggered by various stimuli after destruction of the hard dental tissue surrounding pulp. When microbial incursion happens in pulp, balance between inflammation and reparative process damages. A mild inflammation in pulp is considered reversible pulpitis which can resolve and return to normal pulp after removing the etiology. If etiology is not removed, more immune reaction happens and the balance between damage and repair is broken in dental pulp, leading to uncontrollable inflammation and irreversible pulpitis. If treatment isn’t carried out in time, pulpitis could finally cause pulp necrosis, periapical periodontitis and other serious complications, leading to more medical and economic burden [1].

According to the criteria set by the American Association of Endodontists (AAE), approaches utilized to assess the inflammatory seriousness of pulps is mainly relied on clinical examination and medical history, such as pain characteristics and response to pulp sensitivity tests [2]. Depending on different progress of pulp inflammation, root canal treatment (RCT), vital pulp therapy (VPT) or other surgical endodontic treatment are applied to treat dental pulp of pathological state. However, poor correlations were reported between pulp status and clinical features using histopathological examinations [3]. Additionally, pulp tissue from patients with clinically diagnosed irreversible pulpitis might not present severe inflammation pathologically [4]. Therefore, VPT could be applied to treat even irreversible pulpitis and reported a high successful rate [5]. Histopathological examination is required to determine whether the inflammation state is reversible [6]. However, tooth extraction is required to complete histopathological examination of pulp [7]. Therefore, new diagnostic approach for pulp should be developed to relatively non-invasive determine the pulp state.

At the molecular level, various cell factors are released during pulp inflammation, including cytokines, growth factors, inflammation mediators, antimicrobial peptides, and proteases. Biomarker candidates of pulpitis were screened using bioinformatics analysis of merged datasets [8]. However, understanding and analysis methods of biological characteristics for pulpitis remains limited. The need to merge existing datasets using different methods is increasing to obtain standardized results of pulpitis. WGCNA is applied to integrate gene expression and examine potential mechanisms of gene networks [9]. WGCNA is utilized to study the co-expression modules, genetic network hallmark, and core genes participating in certain oral disease, like Sjögren’s syndrome [10] and periodontitis [11]. Therefore, WGCNA has potential to effectively process data to determine function pathways and promising markers in and identify the hub genes and gene-network signature of pulpitis [12]. Hub genes is genes interacting with other genes in networks and playing an important role in gene regulation and biological processes [13].

As far as we know, WGCNA hasn't been used to process pulpitis data sets. In our research, the investigators first applied WGCNA to study the genetic net hallmark of pulp tissue associated with pulpitis. Furthermore, GO analyses were utilized to explore the potential functions. Additionally, high module group members (MM) methods were carried out to select hub genes and find associated gene set.

## Methods

### Data collection and preprocessing

Two microarray datasets (GSE92681 and GSE77459) were retrieved and acquired from the Gene Expression Omnibus (GEO). GSE92681 including 7 pulpitis and 5 healthy pulp samples from human, using the microarray platform GPL16956 (Agilent045997 Arraystar Human LncRNA Micro-array V3). GSE77459 involved 6 pulpitis samples and 6 healthy specimens from human, using the microarray platform GPL17692 (Affymetrix Human Gene 2.1 ST Array). The information of two datasets were summarized in Table 1.

**Table 1:** Summary of two data sets of pulpitis

GEO ID	GSE92681	GSE77459
Title	Differential expression of LncRNAs and mRNAs in normal and	
Inflamed human pulp	Gene expression profile of pulpitis	
Platform	GPL16956	GPL17692
Number of samples of normal pulp versus inflamed pulp	5 versus 7	6 versus 6
Organism	Homo sapiens	Homo sapiens

### Data processing

After merging two datasets into one file, batch effects were removed using ComBat normalization in SVA package with the normal protocol [14]. Subsequently, the microarray data was transformed into an expression matrix with oligo package [15] and subjected to processing via the limma R/Biological Conductor package [16]. Differentially Expressed Genes (DEG)between samples of normal pulps and pulpitis were screened with the cutoff standards of modified p-value (adj. *P*) < 0.05 and log2 fold change (FC) ≥ 1 using the “limma” R package.

### Establishment of coexpression network

The R package ‘WGCNA’ was utilized to construct the coexpression net based on merged database [9]. The soft-thresholding power was seven as 0.9 was the liminal value of the correlative coefficient, and 50 was selected to be the minimal module size. 0.25 was defined as the liminal value for cut height to integrate possibly alike modules. After selecting 400 genes randomly, the gene network was visualized to be a heatmap based on their cluster dendrogram and Topological Overlap Matrix dissimilarity.

### Enrichment analyses

For the sake of more deeply investigating the genetic role in the module most associated with pulpitis, our team utilized the Database for Annotation, Visualisation and Integrated Discovery (DAVID) to complete GO analyses [17]. *P* < 0.05 was defined as the significance threshold for functional terms. Minimum number of genes was set as 3. The R package “GOplot” was employed to present the outcomes.

### Hub gene determination

Hub genes in the interested module were selected with Cytohubba using high module group members (MM) that indicated a remarkable association with certain clinical characteristics. Top 5 genes were selected for further investigation [18].

### Animal models

The animal assay protocols utilized herein were accepted by The Zhejiang University. 24 male Wistar rats weighing between 270 to 300 g were applied to build pulpitis model, which were stochastically separated into three groups, control group, pulpitis group and iRoot BP Plus group.

The rats were subjected to anesthetization using 50 mg ketamine. Kg^−1^ intraperitoneally. At day 1, all animals except rats in the negative control group had the maxillary incisor pulps exposed by a one-quarter carbide round bur in a highspeed handpiece without water cooling after anesthesia. Temporary restoration material was utilized for the purpose of sealing cavities. In the iRoot group, iRoot BP Plus (Innovative Bioceramix Inc, Canada) was placed on the pulp stumps.

From day 1 to day 4, animals were kept under observation. Intravenously, dipyrone (0.03 mg per 100 g of bodyweight) was applied to treat animals to minimize postoperative discomfort from the day 1 to day 3. At day 5, the rats were injected with intravenously injected dipyrone (0.03 mg per 100 g of bodyweight) and euthanized by CO2 inhalation.

### Sample collection

At day 5, following sacrificing the animals, a hemostat was applied to separate the maxillary incisors from the jaws. Excess soft tissue, bone and periodontal ligament were cleared carefully. The pulp sample was isolated individually, frozen in liquid nitrogen directly and preserved under − 80 °C through the homogenization process.

### RT‑qPCR analysis of pulp samples

Overall RNA was isolated from pulp samples via TRIzol® Reagent (Takara, Japan) as per the protocol provided by the supplier. PrimeScript™ RT Master Mix (Takara, Japan) was applied to convert RNA into cDNA via reverse transcription. a ViiATM7 RealTime PCR System (Applied Biosystem, America) was applied to perform Quantitative RT-PCR assays with SYBR® Premix Ex TaqTM Tool (TaKaRa Biotechnology, Kusatsu, Japan) following the provided instructions. As *HMOX1, LOX, ACTG1, STAT3, GNB5* were considered as hub genes. The detailed sequences of the primers for each gene are listed in Additional file 2: Table S1. The gene *gapdh* was applied as the inner reference. The *ΔΔCT* method was applied to measure the comparative genetic expression levels. Experimental data were expressed as the mean ± SD and assessed by Wilcoxon test. The threshold for statistical significance was set at the level of *P* being 0.05. Therefore, in all cases, *P* < 0.05 was considered statistically significant.

## Results

### Dataset integration and DEGs between pulpitis and normal controls

**Fig. 1:**
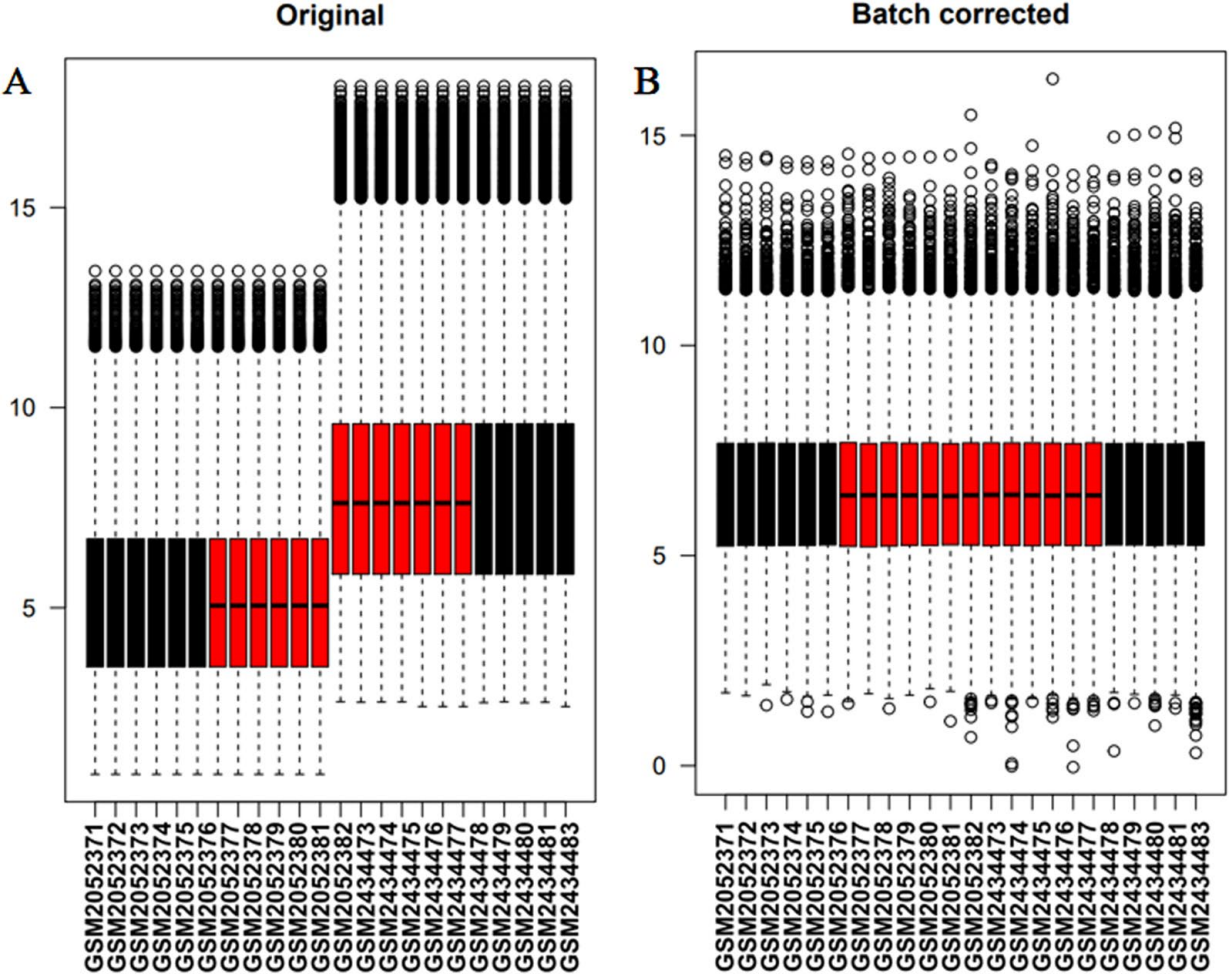
Box plot of data from 24 specimens in the gathered data set prior to and posterior to normalization. The X-axis displays specimens from the data set, and the Y-axis displays normalized intensities. **A** Box plot presenting the expression profiles of the gathered data set prior to normalization; **B** Box plot presenting the expression profiles of the gathered data set posterior to normalization via the SVA package

**Fig. 2:**
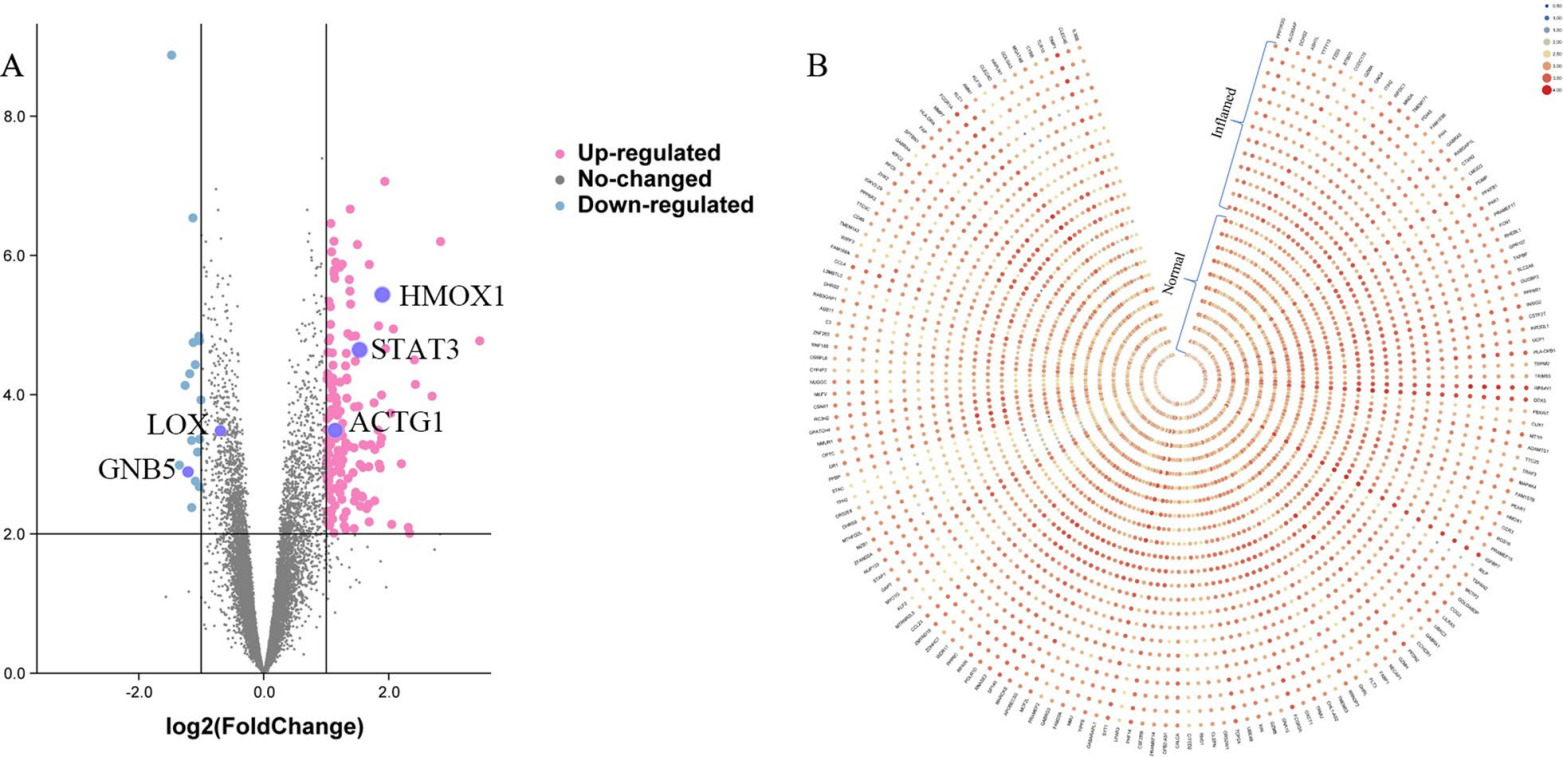
Volcanic plot and heat map of DEGs. **A** Volcanic plot of DEGs in the integrated data set. The X-axis displays the log FC, and the Y-axis displays the -log10 adj.P. The DEGs were determined as per the standards |FC|> 1.0 and adj. *P* < 0.05. The pink and blue circles represent upregulated and downregulated genes in pulpitis, respectively. The grey circles represent non-DEGs between inflammatory and healthy pulps. HMOX1, STAT3, ACTG1, GNB5 and LOX were indicated. **B** Heat map of DEGs in the integrated data set. Blue and red stand for upregulated and downregulated mRNAs in the integrated data set, respectively

The box plot (Fig. 1) showed the expression profiles of gathered data set prior to and posterior to normalization. A principal component analysis (PCA) (Additional file 1: Fig. S1) showed that the pulpitis group can be clearly separated from the control group. Compared with healthy samples, 149 upregulated gene and 17 downregulated genes related to pulpitis were screened, which was presented as a volcano plot (Fig. 2A) and heatmap (Fig. 2B).

### Construction of co-expression modules

**Fig. 3:**
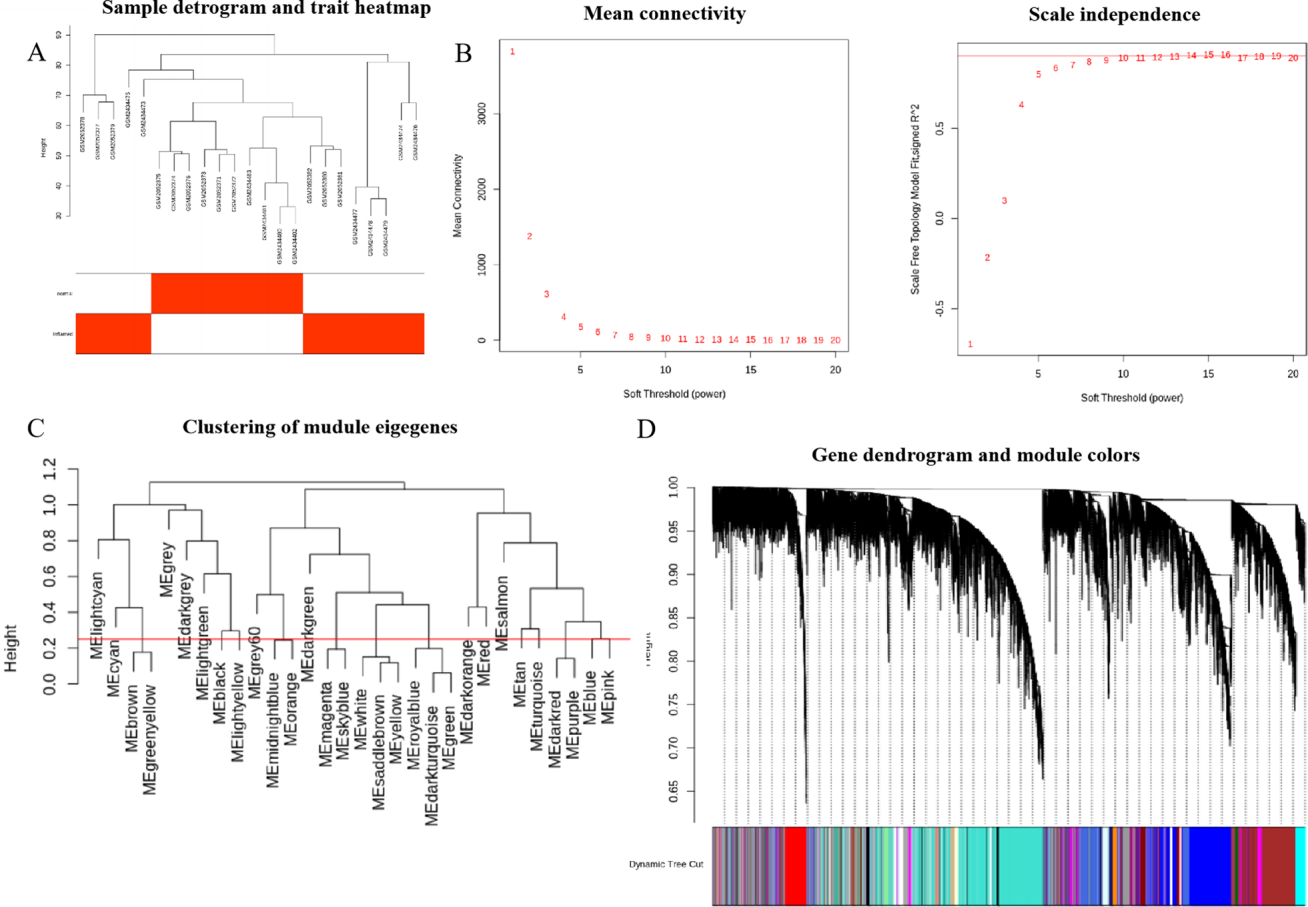
Establishment of weighted coexpression net. **A** Specimen dendrogram and feature heat map. Colors stand for the pulp state (normal and inflamed). **B** Soft threshold selection process. **C** An eigengene dendrogram determined groups of related modules with 0.25 defined as the liminal value for cut height. **D** Every color denotes one specific coexpression module, and branches above stand for genes

After removing an outlier sample, the genes in 23 samples of combined dataset were applied to establish the coexpression network. The outcomes of clustering analyses were displayed by Fig. 3A. A soft-thresholding power was applied to the network topology in relation to mean connectivity and scale independence of the network. A soft-threshold of seven was applied to acquire the nearly scale-free topology as 0.9 was the correlation coefficient threshold (Fig. 3B). As indicated in Fig. 3C, 22 modules were determined where genes displayed alike coexpression features as cut height was 0.25. The module sizes were presented in Fig. 3D.

### Module-trait correlations in pulpitis

**Fig. 4:**
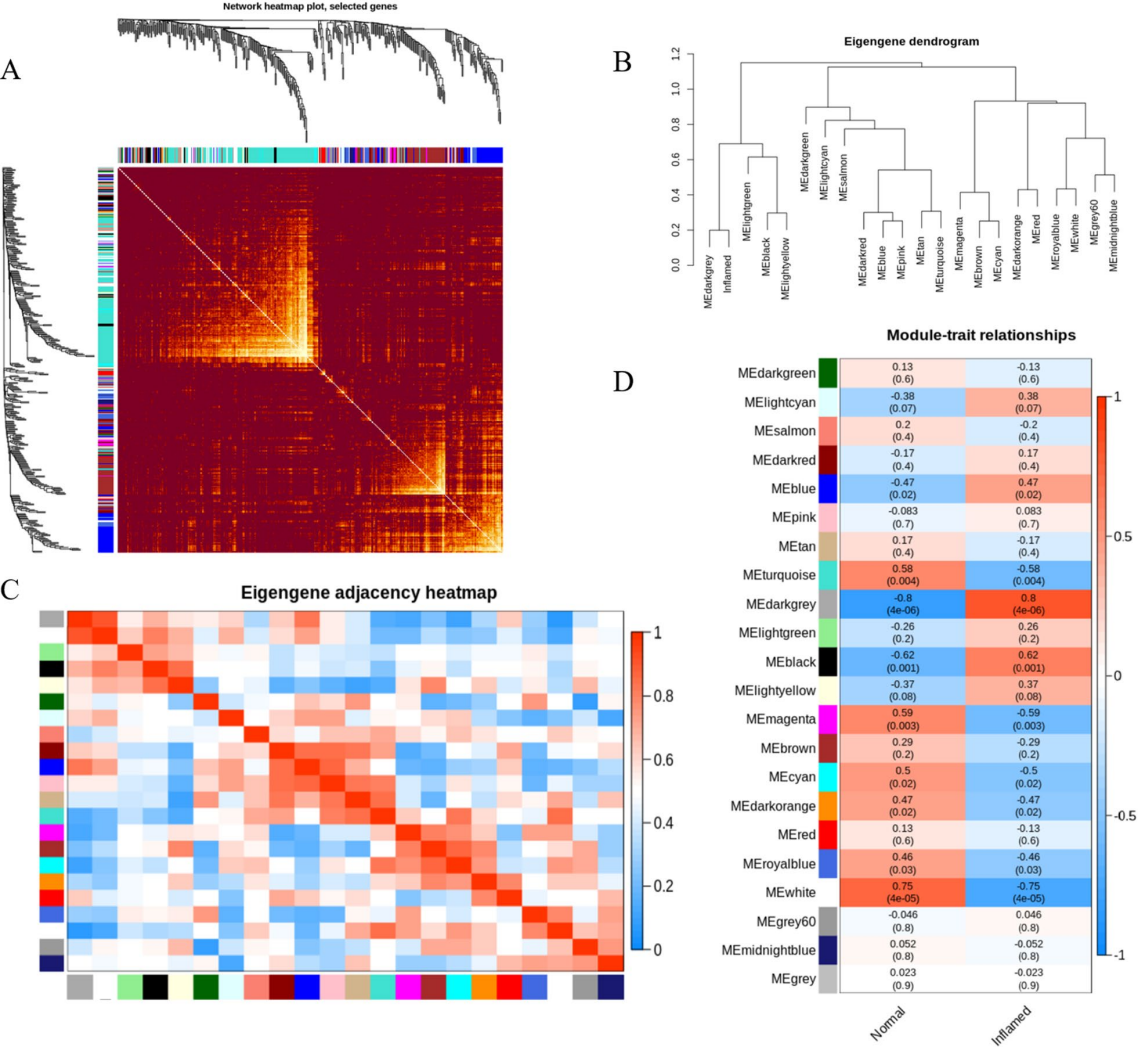
WGCNA module analyses. **A** Interplay of coexpression genes on the basis of TOM dissimilarity and the clustering dendrogram of 400 stochastically chosen genes. The colors of the axes stand for corresponding modules. The intensity of the brown inside the heat map denotes the overlap level of overlap, with a darker brown denoting an elevated overlap. **B** An eigengene dendrogram identified groups of correlated modules. **C** Eigengene adjacency heat map of diverse gene co-expression modules reveal the relationships between them. **D** Heat map of the relationship between pulp state and module eigengenes. Every row denotes a module. Every cell has the correlative coefficients in correspondence to the cell color; blue color denotes negative correlation, while red color denotes positive correlation between module eigengenes and clinical measurements of pulpitis. P-values are described in the brackets

Then we analyzed the interaction associations among these modules. A comparatively high independence level amongst the clusters were suggested by the network heat map of a 400 stochastically chosen genes (Fig. 4A). Additionally, the eigengene dendrogram and eigengene adjacency heatmap both suggested that 22 modules were separated into multiple separate groups (Fig. 4B,C), indicating that coexpression clusters with diverse biofunctions were screened out in the gene network of pulpitis.

By correlating module-specimen eigengenes with clinical features, the relationships of module traits were analyzed to determine significant relationship (Fig. 4D). The colors of the entire modules were stochastically chosen to differentiate between modules. Through highlighting the status feature, the darkgrey module showed the most remarkable association (r = 0.8; *P* < 0.001). Therefore, the darkgrey module was considered as the interest module.

### Functional enrichment analysis in the darkgray module

**Fig. 5:**
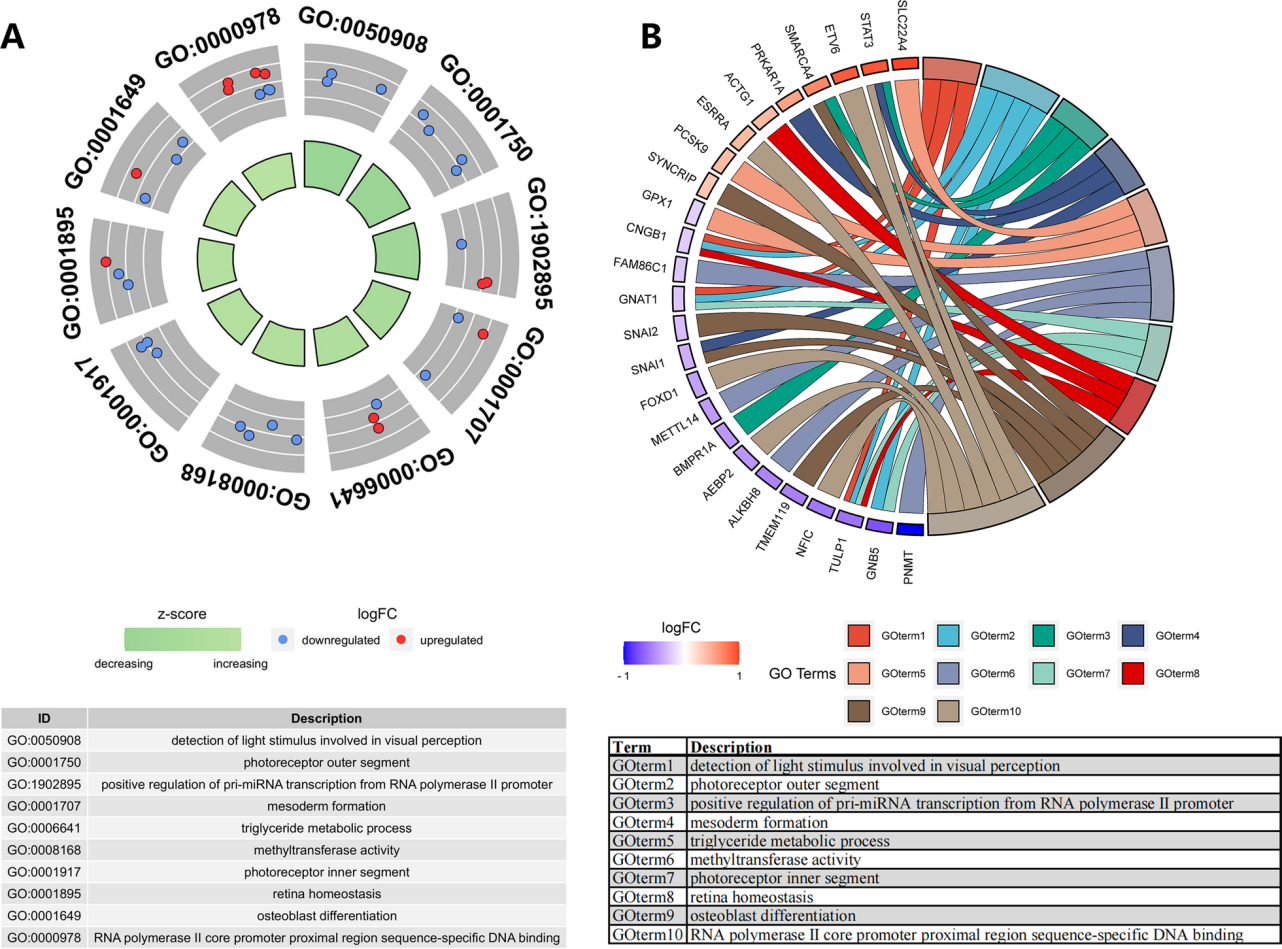
Functional enrichment analyses of darkgrey module. **A** Top 15 terms from GO enrichment analyses. Red and blue dots stand for upregulated and downregulated genes, respectively. The size and color of the sectors denote the adj. P. val and stand score (z-score) of every GO term, separately. **B** Circos plot indicating the relationship between GO terms and genes

GO enrichment assay was completed based on the interest module in circle (Fig. 5A) and circus form (Fig. 5B). The detailed process of GO analysis was shown in Additional file 3. GO analysis suggested that genes in darkgrey module were enriched in triglyceride metabolic process, methyltransferase activity, osteoblast differentiative activity, RNA polymerase II core promotor proximal region sequence-specific DNA binding, positive modulation of pri-miRNA transcription from RNA polymerase II promotor, actin filament binding, microtubule cytoskeleton and so on.

### Module visualization and determination of hub genes

**Fig. 6:**
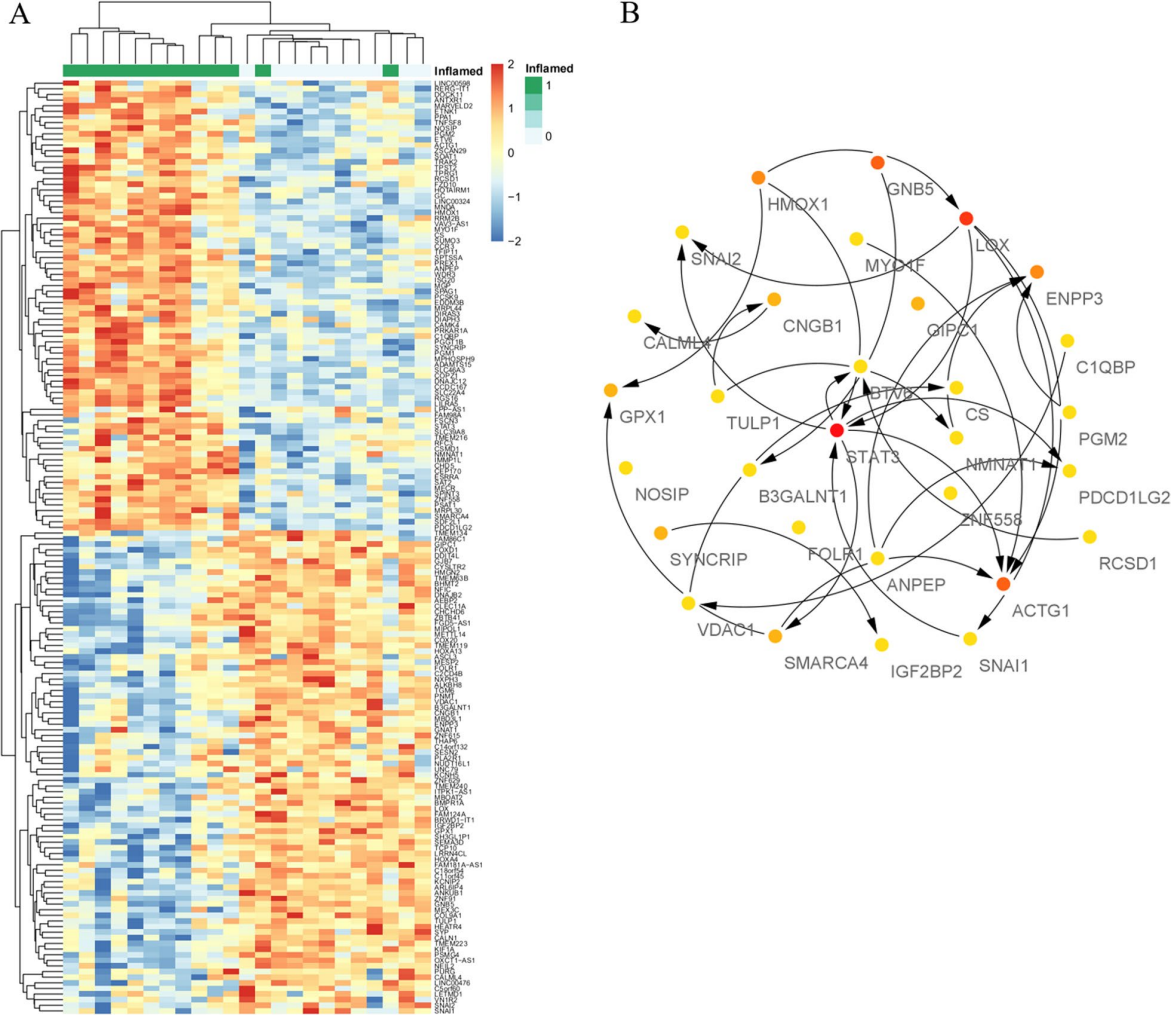
The heat map and hub genes identified from the darkgrey module. **A** The heat map of DEGs in the darkgrey module. **B** Top 30 hub mRNAs in the darkgrey module of pulpitis. The color saturation of a node represents the level of the node in every net

The heatmap for genes in darkgrey module was present in Fig. 6A. Top 30 genes ranked by MM score were visualized by Cytoscape (Fig. 6B). The detailed process of hub gene detection was shown in Additional file 4. The expressing levels of five core genes were identified in the pulpitis rat model according to the MM value. Subsequently, the protein coding genes *HMOX1, LOX, ACTG1, STAT3, GNB5* were selected as the core genes, as they were most negatively or positively correlated modules with pulpitis statistically.

### Validation of hub genes and impact on hub genes expression of pulp capping in pulpitis rat models

**Fig. 7:**
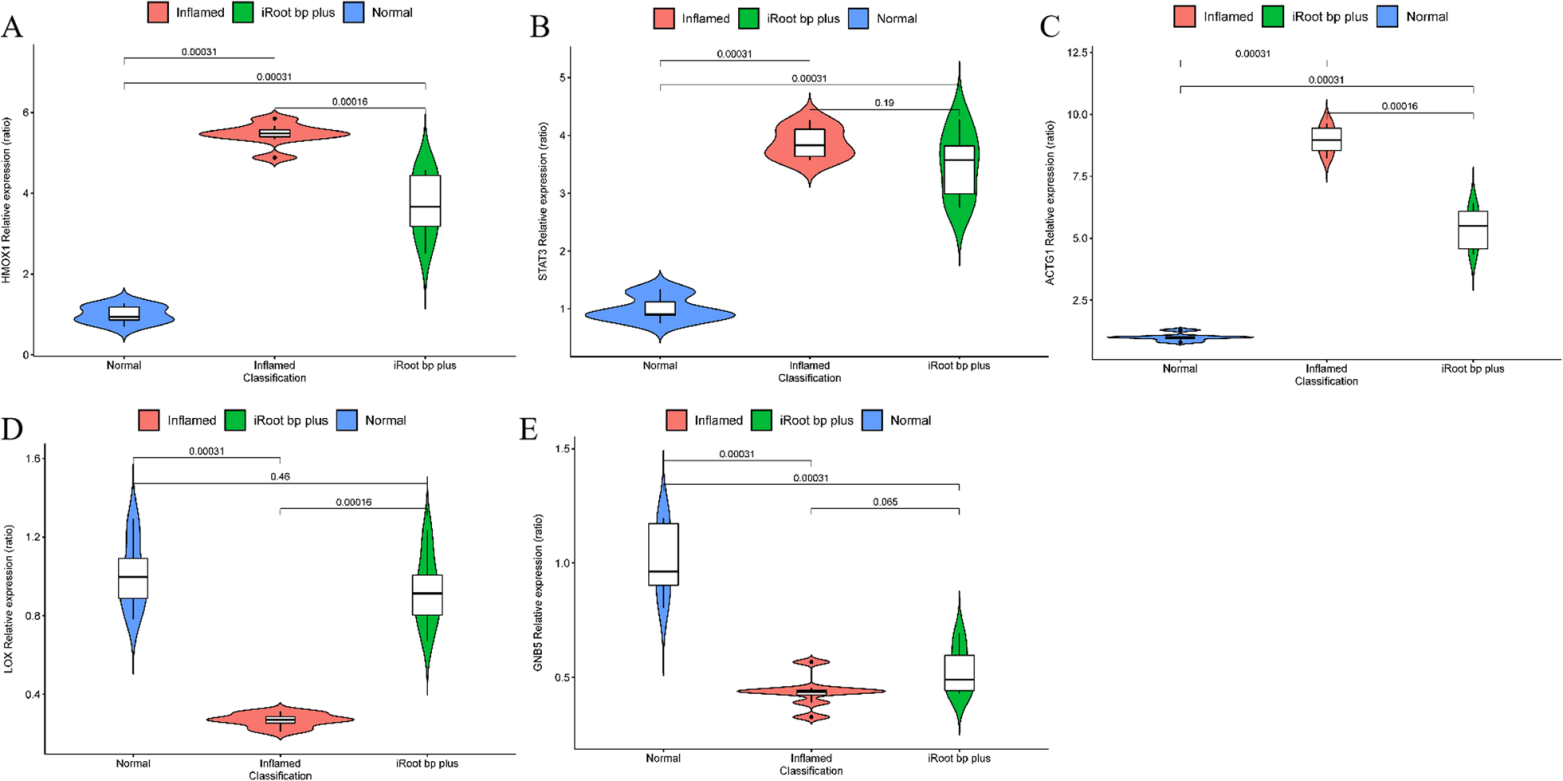
Validation of hub genes and impact on hub gene expression of pulp capping using iRoot BP plus. **A–C** Expressing levels of HMOX1, STAT3, and ACTG1 were considerably upregulated in inflamed pulp and pulp capping using iRoot BP plus reverse the expressing level of HMOX1 and ACTG1. **D**, **E** Expressing levels of LOX and GNB5 were considerably downregulated in inflamed pulp and pulp capping using iRoot BP plus reverse the expressing level of LOX. Wilcoxon test was applied to compare each two group

RT‑PCR was performed to validate the hub genes and analyze the changes of hub gene in pulp after iRoot BP Plus capping. The expressing level of *HMOX1, LOX, ACTG1, STAT3, GNB5* were present in Fig. 7. The expressing levels of *STAT3, HMOX1* and *ACTG1* were higher in pulp of inflamed group than that of normal group significantly, but only levels of *HMOX1* and *ACTG1* were lower in pulp of iRoot group than that of Inflamed group. The expressing levels of *LOX* and *GNB5* were lower in pulp of inflamed group in contrast to normal group significantly. Only *LOX* was higher in pulp of iRoot group than that of inflamed group.

## Discussion

In previous stuty, intersections of PPI network nodes and pulpitis-related genes were screened out in a merged dataset [19]. WGCNA was performed to analyze two data respectively and identify the significant gene modules in each dataset [12], which demonstrates the genetic and epigenetic mechanisms of irreversible pulpitis by revealing the ceRNA network. However, the present paper is the first to use WGCNA to build the pulpitis-related gene-network in a merged data. Pulpitis-related gene co-expression networks was built with WGCNA method. Key gene coexpression module and core genes were considered to be related to pulpitis. The outcomes acquired herein can offer fresh enlightenment pertaining to the molecule-level causal link of the developmental process of pulpitis.

As the development of biomaterials, VPT are increasingly being utilized for preserving vital pulp and increasing the longtime survival [20]. However, accurately diagnosing the inflammation degree of pulps is usually challenging for clinicians [20]. The failure of VPT is always due to the absence of accurate diagnosing tools. As histology features of the inflammation do not correspond with the clinical signs of pulpitis, advanced diagnostic approach should be developed to compensate for the deficiency of traditional diagnostic approach based mainly on clinical manifestation [2, 21]. We screened out hub genes using WGCNA method and verified that the hub genes expressed differentially in pulpitis model, indicating that the genes could have potential future applications as a biomarker for diagnosis.

Pulpitis is tightly associated with host immune and inflammatory response in relate to molecular factors [22]. Opportunistic infection by dental microorganisms like Streptococcus species accounts for the onset and development of pulp Inflammation, which is caused by caries, trauma and other possible stimulations [23]. Immune reaction is triggered immediately in response to irritation and pathogen in pulp to hinder the infection propagation, launch reparative process and stimulate relevant signaling [23]. Unfortunately, irreversible pulpitis, pulp necrosis or even periodontitis occur if the balance between pulp reparative process and inflammatory response is broken [24]. The judgement on the degree of inflammation is crucial for treatment selection. Several cytokines and genes have been considered as potential diagnostic markers for pulp inflammation [25]. However, to the best of our knowledge, there is still no biomarker that could be applied to accurately diagnose degree of pulpitis alone. To partially address this issue, multiple predictors were applied to be biomarker to predict the pulpitis in present study.

In this study, we used 13 inflamed vs 11 normal microarray datasets to carry out WGCNA. The original research containing above two data sets, finished by Galicia et al. [26] and Huang et al. [27], revealed the diversities of genetic expression profiles between inflamed and normal pulps, and indicated that pulpitis was related to differential genetic expression, which was associated with immune system process and cardiac ventricle development [27], as well as Beta2 integrin cell surface interactions [11]. The important differences between the modules in this paper and the outcomes from the research of Galicia et al. [26] and Huang et al. [27] was that our research more systematically applied analysis approach, WGCNA, determining the darkgray module as the most relative module with pulpitis with high enrichment in methyltransferase activity, triglyceride metabolism and positive modulation of pri-miRNA transcription from RNA polymerase II promotor. Conventional microarray DEGs analysis would be unable to obtain in-depth analysis and results.

In our paper, an overall 22 coexpression modules were acquired via WGCNA analyses. Amongst them, the darkgray module was the most important one participating in pulpitis. Enrichment analyses suggested that the genes in the darkgray module were mainly linked to pathways correlated with methyltransferase activity, triglyceride metabolism and positive modulation of pri-miRNA transcription from RNA polymerase II promotor. Pulpitis is an inflammatory event of dental pulps, which is induced by the reaction to external stimuli. The reaction is related to many substantial cellular and molecular activities, it was reported that trimethylation of lysine 4 histone 3 (H3K4me3) was involved in differentiation of odontogenic progenitors in pulpits process, which agrees with our findings [28]. Additionally, evidence supports that triglyceride metabolism and positive modulation of pri-miRNA transcription from RNA polymerase II promotor are found to be related to inflammatoty responses [29, 30]. However, to the best of our knowledge, there are currently no studies investigating the role of triglyceride metabolic process and positive regulation of pri-miRNA transcription from RNA polymerase II promotor in pulpitis. Triglyceride metabolic process can lead to activation of NFK beta, VCAM-1 and other inflammation mediation factors [29] and the decrease in triglyceride levels are related to pulpal diseases in diabetic rats [31]. RNA polymerase II promotor is highly connected to inflammation process in terms of macrophage gene expression as 60% of inflammation macrophagus transcriptome could be modulated predominantly via RNA polymerase II promotor proximal promotor pausing and releasing [30]. Therefore, deeper investigation of darkgray module is needed to precisely explain the causal links related to the developmental process of pulpitis.

Hub genes screened in present study have potential to be biomarkers of dental pulp blood analysis [32]. Due to its advantages of ease-of-use and non-invasiveness, dental pulp blood analysis is a promising method for the molecular diagnosis of pulpitis, which could reflect the pathophysiologic conditions of dental pulp in inflammation [33]. Our study could help to achieve the goal to develop a non-invasive, low-cost, chair-side rapid method of pulpitis diagnosis.As darkgrey modules showed the highest levels of association with pulpitis, the core genes in the module were selected. The top 5 genes in darkgrey module were screened as the intramodular core genes of pulpitis. *HMOX1, LOX, ACTG1, STAT3, GNB5* were selected as hub genes. HMOX1 is an important factor of obesity, tissue dysfunction, intestinal inflammation and metabolic disturbances [34]. It is considered as cell protective enzyme and can promote cytokine secretion, suppress phenotypic maturation in the immune effector cells, and enhance cytokine production [35]. However, to our knowledge, *HMOX1* has not been studied in pulpitis yet. STAT3 is a cellular signal transcriptional factor related with the regulation of many cellular activities, which contributes to anti-inflammatory process and repairing of damage tissues [35]. Moreover, *STAT3* has two roles in tumorous inflammatory events and immunoactivity via stimulating pro-oncogenesis inflammation paths, like interleukin-6 (IL-6)-GP130-Janus kinase (JAK) pathway, nuclear factor-kappa B (NF-kappa B) pathway, and opposing STAT1- and NF-kappa B-mediated T helper 1 anticancer immunoresponses [36]. In line with our study, IL6/JAK/STAT3 signaling pathway was found to be associated with pulpitis [8]. In present study, we found *HMOX1* and STAT3 were significantly higher in pulp of Inflamed group than that of normal group, and lower in pulp of iRoot group than that of Inflamed group, which indicated that *HMOX1* and *STAT3* are important inflammation factors in pulpitis, and the expression level of them could be reversed by vital pulp treatment. Modulation of *LOX* expression regulates recombination human VEGF to promote cellular growth, odontogenic potential and in vitro revascularization [37]. LOX-mediated organization of collagen fibril in extracellular matrices is vital for regulating odontoblastic differentiative activity of human dental pulp cells [37]. Therefore, regulation of *LOX* expression is important in repair of damaged tissue in pulp. In our study, *LOX* was inhibited in inflamed pulp, and pulp capping of iRoot BP plus increased the expression of *LOX*, thereby promoting angiogenesis and odontogenesis, which might promote tissue repair. *ACTG1* is a key structural gene that helps the construction of cell cytoskeletons, and exerts an effect on biofunctions like division, migratory ability, and vesicle trafficking. Wu et al. [38] found *ACTG1* regulates the proteins of inflammation-related pathways, which indicated *ACTG1* might serve as a new biomarker and treatment target of inflammation. *GNB5* has widespread expression and is capable of encoding guanine nucleotide-binding protein sub-unit beta-5 (Gβ5). Central nervous system G-protein signal transmission is downregulated by Gβ5 through the interplay with G-protein–coupled acceptors. *GNB5* knockdown mice have damaged neurological, retinal, and cardiac functions [39]. However, to our knowledge *ACTG1* and *GNB5* have not been studied in pulpitis yet. In present study, changes of expression levels of *ACTG1* and *GNB5* were observed which indicated they may be associated with the inflammation status of pulp. More in depth researches are needed to interrogate the effects of *ACTG1* and *GNB5* on pulpitis.

Pulp-capping agents ought to harbor antibacteria, nontoxicity, antiinflammation and excellent sealing abilities, and ought to induce dentin mineralization [40]. The pulp capping using Mineral Trioxide Aggregate (MTA) could reduce inflammation cells, decrease vascular density, and regulate IL-6 expression in mandibular first molars from Wistar rats [41]. iRoot BP Plus has shown similar properties and result with MTA in the pulpectomy in dog teeth [42] and displays a superior clinical handling property compared with MTA. Morepver, iRoot BP Plus possesses splendid compatibility, the capability of eliciting odontoblast differentiation and mineralization [43]. It's deemed as an appropriate alternative for calcium hydroxide in the pulpectomy of permanent teeth [43]. Clinically, iRoot BP Plus is quite prospective as a pulp-blocking agent. Therefore, we used iRoot BP plus as blocking agent to construct the model for VPT in the treatment of pulpitis. In present study, pulp capping of iRoot BP plus could successfully rescue the inflammation state and reverse the expression level of some hub genes (*HMOX1, ACTG1, LOX*). It indicated that pulp capping using iRoot BP plus could regulate inflammation and promote the ability of angiogenesis and odontogenesis. However, in our study, the levels of *STAT3* and *GNB5* were not reversed, and these two genes were less studied in pulpitis. Therefore, the role of *STAT3* and *GNB5* in pulpitis and material modification to regulate these two genes should be further investigated.

## Conclusion

WGCNA was completed based on a pulpitis data set. Amongst the 22 modules, the darkgray module was determined as the most pivotal module for pulpitis, from which 5 core genes, A *HMOX1, LOX, ACTG1, STAT3* and *GNB5*, were selected, and assumed to exert pivotal effects on the pathophysiologic causal links of pulpitis. The darkgray module was determined to be related to methyltransferase activity, triglyceride metabolism and positive modulation of pri-miRNA transcription from RNA polymerase II promotor. Those outcomes could foster further experiment researches on the function of the genes in pulpitis pathogenesis, which haven't been described yet. Moreover, the identified genes may be considered new treatment targets for the therapies of pulpitis, and help to further reveal the latent causal links regarding pulpitis.

RT-PCR proved the differences in expression levels of *HMOX1, LOX, ACTG1, STAT3, GNB5* in inflamed dental pulp compared to healthy dental pulp. Pulp capping reversed the expression level of *HMOX1, LOX, ACTG1*. More modification of the material properties is required to regulate the expression level of *STAT3, GNB5* and restore the pulp to its normal physiological state.

## Supplementary Information

**Additional file 1.** **Figure S1**. PCA results after batch effect removal showed that the inflamed group can be clearly separated from the control group.

**Additional file 2.** **Table S1**. The detailed sequences of the primers for each gene.

**Additional file 3.** Detailed process of GO analysis.

**Additional file 4.** Detailed process of hub gene detection.

## Data Availability

The datasets used for the current study are available from the corresponding author on reasonable request.
