# Supplementary material for: Identification of key module and hub genes in pulpitis using weighted gene co-expression network analysis
Source: BMC Oral Health. 2023 Jan 2;23:2. doi: 10.1186/s12903-022-02638-9 (PMC9808982; doi:10.1186/s12903-022-02638-9)
Supplement: Supplementary file 1 — Additional file 1. Figure S1. PCA results after batch effect removal showed that the inflamed group can be clearly separated from the control group. [file 12903_2022_2638_MOESM1_ESM.docx]

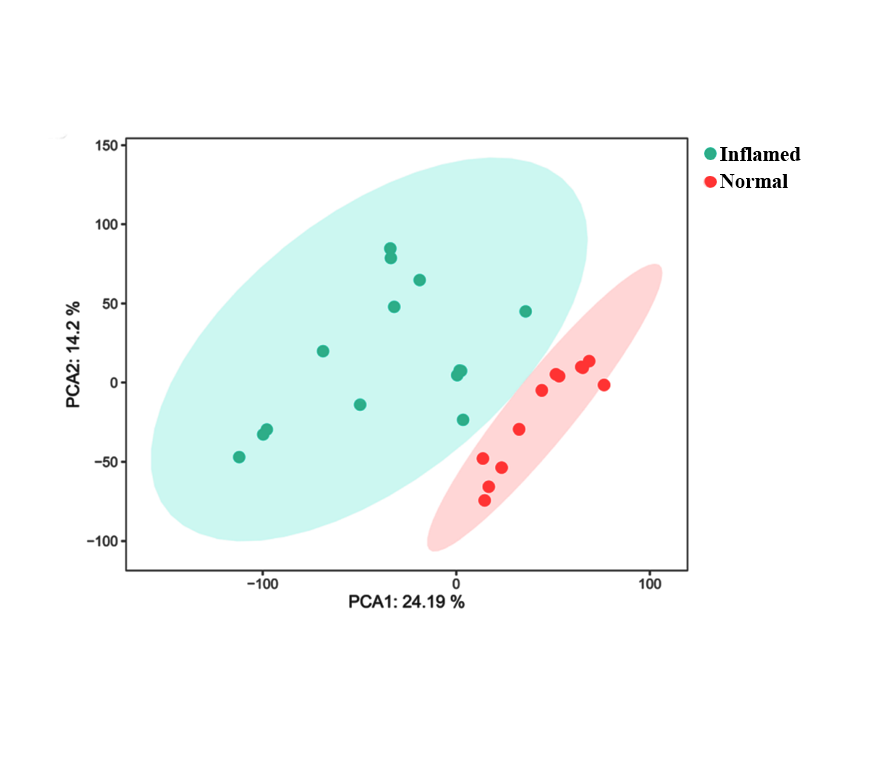


Figure S1. PCA results after batch effect removal showed that the inflamed group can be clearly separated from the control group.
