# Supplementary material for: Identification of key module and hub genes in pulpitis using weighted gene co-expression network analysis
Source: BMC Oral Health. 2023 Jan 2;23:2. doi: 10.1186/s12903-022-02638-9 (PMC9808982; doi:10.1186/s12903-022-02638-9)
Supplement: Supplementary file 2 — Additional file 2. Table S1. The detailed sequences of the primers for each gene. [file 12903_2022_2638_MOESM2_ESM.docx]

| **Gene** | **Forward primer (5' -> 3')** | **Reverse primer (5' -> 3')** |
| --- | --- | --- |
| HMOX1 | CGAGGAAAATCCCAGATCAGC | CATCACCAGCTTAAAGCCTTCT |
| LOX | CGATTTGCCTGTACTGCACAC | CTGGGGTTTACACTGACCTT |
| ACTG1 | ACCACTTCTTTTCTTGCCAGT | ACTCAAGGCAAGTAACAACCC |
| STAT3 | AATACCATTGACCTGCCGAT | CTTCCCCGTTATTTCCAAACTGC |
| GNB5 | ACGTGTCGCCTCTATGACC | AGAAATCCACGCTTGATGCTC |
| GAPDH | TGATTCTACCCACGGCAAGTT | TGATGGGTTTCCCATTGATGA |

**Table S1.** The detailed sequences of the primers for each gene
